# Pathological Manifestations of Gluten-Related Neuro-Psychiatric Disorders and the Impact of Gluten-Free Diet in a Pediatric Age Group: A Systematic Review

**DOI:** 10.7759/cureus.47062

**Published:** 2023-10-15

**Authors:** Prajwala Nagarajappa, Sree Mahathi Chavali, Maneeth Mylavarapu

**Affiliations:** 1 Department of Pathology, Mysore Medical College and Research Institute, Mysore, IND; 2 Department of Pediatrics, GSL Medical College, Rajahmundry, IND; 3 Department of Public Health, Adelphi University, Garden City, USA

**Keywords:** systematic review, celiac disease, pediatric age group, neuropsychiatric manifestations, gfd, gluten

## Abstract

Gluten, as a term, causes unease among a vast majority of the population. The reason is the body's inability to process gluten, causing various pathological manifestations. While celiac disease is predominantly a gastrointestinal disease, it also has various extra-intestinal manifestations. Many children receive diagnoses of idiopathic neuropsychiatric symptoms such as epilepsy, attention-deficit hyperactivity disorder (ADHD), restless leg syndrome (RLS), and peripheral neuropathy without ever finding the root cause. A majority of these cases may be associated with celiac disease if only their antibody titers and other appropriate investigations were conducted. The treatment of these manifestations may be eliminated or at least controllable with dietary modification to a gluten-free diet (GFD). In this paper, we will discuss the pathology of celiac disease and the impact of GFD on the neuropsychiatric aspects of this disease, which is of higher prevalence in the pediatric population.

A comprehensive literature search was conducted in prominent databases, namely PubMed and Google Scholar, to include studies that provided individual-level data on the neuropathological manifestations and the impact of a GFD on extra-intestinal manifestations of celiac disease. The research protocol was registered in the PROSPERO database (International Prospective Register of Systematic Reviews) with the registration ID: CRD42023415100. Based on the inclusion and exclusion criteria, we included prospective studies, observational studies, and case reports on pediatric patients with biopsy-proven celiac disease, serologically positive celiac disease, celiac disease with neuropsychiatric manifestations, and studies reporting the impact of GFD. After a rigorous quality assessment to remove the risk of bias, we finally included 20 studies to be discussed.

In 6 (30%) studies, patients with neuropsychiatric manifestations had positive serology findings and a relatively higher grade of biopsy results. Seven studies discussed the positive impact of GFD. Five of these seven studies reported statistically significant results (p ≤ 0.001). Our study suggests that gluten plays a role in the severity of neuropsychiatric manifestations of celiac disease. Considering the results of our study, we can see that GFD does impact the prognosis of the disease. Neuropsychiatric findings without gastrointestinal manifestations are more common in the pediatric age group. We have clear evidence that several neurological conditions (neuropathy, ADHD, epilepsy, and RLS) have not only a significant association with gluten but can also potentially benefit from GFD. Thus, screening, with a combination of serological, biopsy, and imaging techniques, must be adapted into the guidelines for early detection and induction of GFD. Furthermore, studies should aim at introducing GFD in the pediatric population as a mode of primary prevention. In conclusion, our review underscores the importance of gluten while dealing with idiopathic neurological conditions in children and hopes to shed light on this commonly misdiagnosed and easily manageable disease.

## Introduction and background

Gluten-related neurological disorders have emerged as a complex and enigmatic area of study. While symptoms are predominantly intestinal, it is also known to cause a multi-systematic disease [1]. Many hypotheses have been proposed, such as immune-mediated enteropathy, malabsorption sequelae, and vasodilation due to pro-inflammatory cytokines [2]. This leaves some uncertainty with both medical professionals and parents of affected children alike, which we have attempted to understand and address in this review. From unexplained behavioral changes to debilitating neuropathies, these disorders pose significant challenges to the well-being of children. 

**Figure 1:**
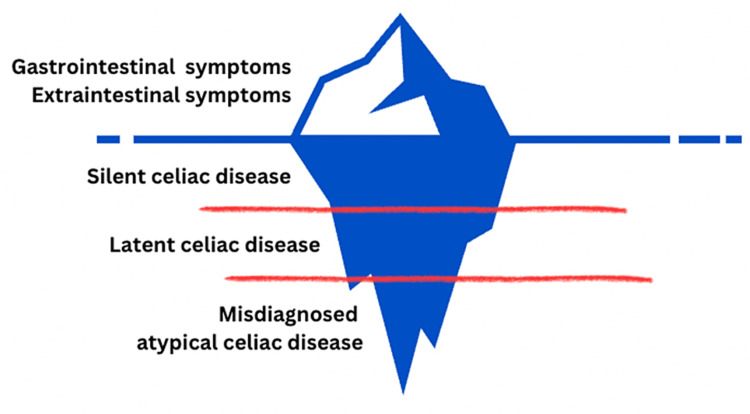
Iceberg Phenomenon of Celiac Disease Image credit: Prajwala Nagarajappa

The global prevalence of celiac disease (CD) is estimated to be 1%. Among these, serologically confirmed and biopsy-proven cases were 1.4% and 0.7%, respectively. Europe (0.8%) and Oceania (0.8%) have the highest prevalence, and South America (0.4%) the lowest, according to a recent population-based study. Surprisingly, in children, a higher prevalence was found in various European countries and North America at 1% of the population. This could be due to increased antibody screening or geographic and ethnic variations such as susceptibility to HLA DQ2/8 [1,3]. A significant portion of these children, in particular, present with atypical symptoms, of which the neuropsychiatric manifestations are discussed in this paper. Hence, there is a heavy burden of misdiagnosis, which contributes to the iceberg phenomenon [4], a representation of which has been depicted in Figure 1.

In this article, we aim to shed light on the pathogenesis underlying the most common of these neurological manifestations in children in order to unravel the intricate web of gluten's impact on the developing pediatric brain. Furthermore, we explore the potential of a gluten-free diet (GFD) as a therapeutic approach. GFD is a widely known dietary modification that excludes gluten, gliadin, and its related products from one's intake. Through various pathophysiological aspects, it can stop the progression of and even resolve some of these symptoms, offering a glimmer of hope for the affected children and their families.

## Review

Methodology

Registration and Protocol

We conducted our systematic review as per the Preferred Reporting Items for Systematic Reviews and Meta-Analyses (PRISMA) guidelines [5] and registered the protocol in the International Prospective Register of Systematic Reviews (PROSPERO) with the registration number CRD42023415100. Refer to the protocol for a detailed description of our objectives and protocol.

Search Strategy

Two databases, namely PubMed and Google Scholar, were selected. We incorporated the building block technique to find relevant studies based on keywords and Medical subject heading (MeSH) keywords. The number of hits for each is recorded in Table 1. These were then subjected to filters based on the exclusion criteria. On PubMed, these were original articles published in a language other than English, studies published more than 10 years ago, and studies including adult participants (≥18 years). On Google Scholar, filters were applied to exclude studies published more than 10 years ago. Fifty-nine studies from PubMed and 2430 studies from Google Scholar were screened based on abstract, and 2366 irrelevant studies were excluded. One hundred and twenty-three studies were then sought for data extraction.

**Table 1:** Search strategy and MeSH keywords

S.No.	Keywords	PubMed/MEDLINE	Google Scholar
1.	[Gluten] OR [Gliadin] OR [Coeliac disease] OR [Immune Enteropathy] OR [Gluten free diet]	Before filters - 18,596; After filters - 1,376	Before filters - 22,000; After filters - 20,200
2.	[Gluten] OR [Gliadin] OR [Coeliac disease] OR [Immune Enteropathy] OR [Gluten free diet] AND [Neurological manifestations] OR [Optic Neuritis] OR [Seizures] OR [ataxia] OR [neuropsychiatric manifestations]	Before filters - 58,474; After filters - 21,291	Before filters - 6,420; After filters - 3,280
3.	[Gluten] OR [Gliadin] OR [Coeliac disease] OR [Immune Enteropathy] OR [Gluten free diet] AND [Pathology] OR [Histopathology] OR [Pathophysiology] AND [Neurological manifestations] OR [Optic Neuritis] OR [Seizures] OR [ataxia] OR [neuropsychiatric manifestations]	Before filters - 128,444; After filters - 43,643	Before filters - 4,780; After filters - 2,430
4.	(("Celiac Disease/complications"[Mesh] OR "Celiac Disease/genetics"[Mesh] OR "Celiac Disease/pathology"[Mesh])) AND ("Nervous System Diseases/diet therapy"[Mesh] OR "Nervous System Diseases/etiology"[Mesh] OR "Nervous System Diseases/pathology"[Mesh] OR "Nervous System Diseases/therapy"[Mesh])	Before filters - 239; After filters - 59	N/A

*Eligibility Criteria* 

The inclusion criteria are as follows: (1) population (ages between 1 and 18 years); (2) GFD with respect to neurological and psychiatric manifestations of CD; (3) case reports pertaining to information regarding neurological and psychiatric manifestations; (4) both gross and histopathological evidence were considered. In addition, only human studies and those in the English language published in the last 10 years were reviewed.

The exclusion criteria were (1) studies that included the above search terms, but which did not narrow the diet intervention to neurological and psychiatric manifestations; (2) studies that included other related diagnoses (e.g., inflammatory bowel disease/IBD, Crohn's disease, and CD) and did not report the results that included the search terms; (3) reviews and other gray literature; and (4) articles that did not contain data relevant to GFD and neurological/psychiatric manifestations. 

Selection of Studies

**Figure 2:**
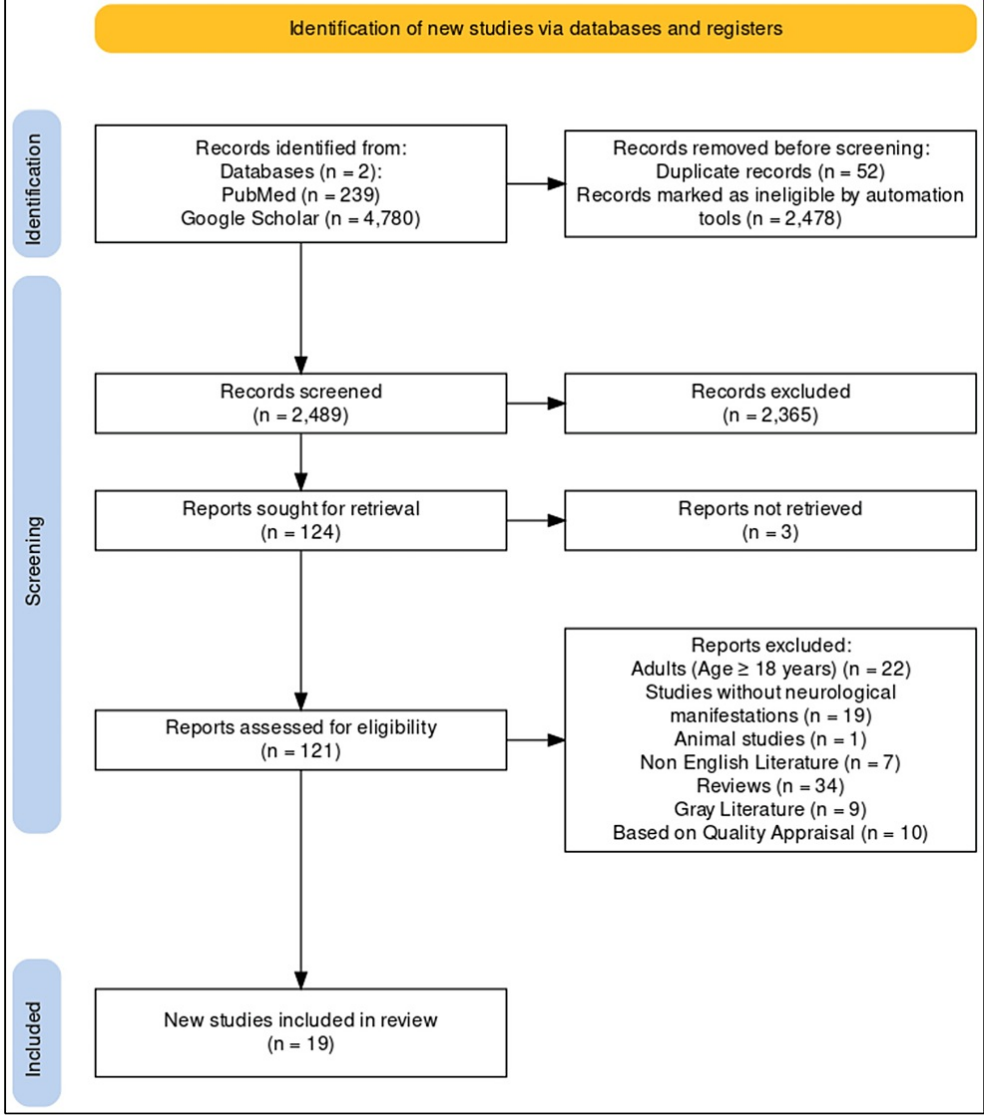
PRISMA flow diagram PRISMA, Preferred Reporting Items for Systematic Reviews and Meta-Analyses.

We reviewed the literature obtained as depicted in Figure 2 [5]. The retrieved articles were imported into the citation manager Endnote and screened based on inclusion and exclusion criteria as well as quality assessment tools. The review was performed by dividing the dataset into three sets, namely A, B, and C. Dataset A was independently reviewed by SC and MM, and disagreements were resolved by PN. Similar steps were taken for datasets B (MM and PN reviewed, disagreements resolved by SC) and C (PN and SC reviewed, disagreements resolved by MM). 

Quality Assessment

The risk of bias in the individual studies was determined by using specific scales for each study. The CARE checklist was used for quality assessment for case reports and case series. For observational studies, it was the Strobe criteria, and for randomized controlled trials, the Cochrane risk assessment tool. Studies with more than 70% scores on respective checklists were considered enough quality to be included in the study.

Results

Number, Type, and Characteristics of Studies

After an investigation of the 121 retrieved articles, each underwent thorough screening on the basis of quality assessment tools, and inclusion and exclusion criteria. Seven articles were not originally published in English, one article was an animal study, nine were gray literature, and 10 studies were excluded based on quality appraisal. Thirty-four studies were review articles, 22 studies did not meet the age criteria, and 19 studies did not report any neurological manifestations. After the above-mentioned exclusions, 19 articles qualify to be a part of the study. An overview of these studies is outlined in Table 2.

**Table 2:** A general overview of the included studies ADHD, Attention-Deficit Hyperactivity Disorder; Anti-TG 6, Anti-Transglutaminase 6; CD, Celiac Disease; CEC, Celiac disease Epilepsy Cerebral calcification complex; CSHQ, Children’s Sleep Habits Questionnaire; DRE, Drug-Resistant Epilepsy; EEG, Electroencephalogram; DTR, Deep Tendon Reflexes; GFD, Gluten-Free Diet; GTS, Gilles de Tourette Syndrome; OCD, Obsessive Compulsive Disorder; OSA, Obstructive Sleep Apnea; PCC, Posterior Cerebral Calcifications; RLS, Restless Leg Syndrome; tTG, Tissue Transglutaminase; QOL, Quality Of Life.

Author	Year	Type of Study	Characteristics of Study
Aksoy et al. [6]	2016	Cross-sectional study	Of 65 CD children who underwent neurological examination, 16 (24.6%) were found to have abnormal findings.
Ferlazzo et al. [7]	2019	Cross-sectional study	60 participants were included in the study, which was divided into four groups. Group 1 (CD, PCC and epilepsy (CEC)) 1/9 (11%), Group 2 (PCC and epilepsy without CD) 2/9 (22%), Group 3 (epilepsy of unknown origin) 0/20, and Group 4 (healthy controls) 3/22 (13.6%) had anti-tTG6 positivity.
Işikay and Kocamaz [8]	2015	Cross-sectional study	40/297 (13.5%) children with known CD were found to have a neurological abnormality. Severe disease (MARSH 3b) was found in these children compared to children without neurological manifestations (MARSH 3a). These results were statistically significant.
Işikay et al. [9]	2018	Cross-sectional study	Although RLS prevalence was similar in the CD group (3.5%) compared to the control group (3%), the CD group had a statistically significant association with regard to the severity of illness and time of onset (early presentation).
Swartwood et al. [10]	2021	Cross-sectional study	A Chart review was performed on pediatric patients with both epilepsy and CD and compared with a control group with epilepsy only. DRE was more prevalent in pediatric patients with biopsy-confirmed CD and positive tTG-Ab compared with the control group. Adherence to a GFD in combination with antiseizure medications appears to reduce the seizure burden for those with CD and DRE.
Gungor et al. [11]	2013	Case-control study	4 out of 362 patients with ADHD (1.1%) and three out of 390 controls (0.8%) were found tTG IgA positive.
Sel et al. [12]	2017	Retrospective study	Out of 117 atypical CD patients, 8 (6.83%) were found to have neurological abnormalities. 5/8 were evaluated on GFD, out of which four children had a positive outcome.
De Leo et al. [13]	2018	Retrospective cohort	Of 274 CD children and 124 controls studied, a significant difference in anti-TG6 levels was detected between the groups (p < 0.004).
Rodrigo et al. [14]	2018	Prospective study	29 children with GTS were assessed for a period of one year on GFD, upon which a statistically significant reduction was found in tics, and OCD symptoms, and general improvement in QOL was found.
Suzer Gamli and Keceli Basaran [15]	2022	Prospective study	103 known CD patients were evaluated with sleep scores while on GFD. 74/103 (71.8%) had a CSHQ score above the clinically significant cutoff pre-GFD, which reduced to 40/103 (38.8%) six months post-GFD. These results were statistically significant.
Yerushalmy et al. [16]	2018	Prospective study	Children aged 2-18 years were prospectively recruited before the initiation of a gluten-free diet. Children with celiac disease had fewer OSA-related symptoms than controls, but the degree of improvement following the initiation of a gluten-free diet was significantly higher.
Işikay et al. [17]	2015	Prospective study	EEG findings were positive for epileptiform activity in 4/43 (9.3%) newly diagnosed CD, 2/132 (1.5%) formerly diagnosed CD, and 1/99 (1%) controls. tTG levels positively correlated with epileptiform activity in both sleep and awake EEGs. These results were statistically significant.
Bashir et al. [18]	2019	Case report	A 16-month-old girl child presenting with vomiting and regression of motor skills was diagnosed with CD. Implementation of GFD has led to improvement in gastrointestinal and neurological symptoms.
Boskovic and Stankovic [19]	2014	Case report	A 15-year-old girl with known CD presented with acute weakness and a pricking sensation in her lower limbs after the accidental introduction of gluten in her diet. After extensive evaluation, she was found to develop demyelinating peripheral neuropathy. Spontaneous improvement in the clinical picture was seen after restarting GFD.
Ching et al. [20]	2015	Case report	A 4-year 5-month-old female with a clinical history of gluten sensitivity presented with recurrent episodes of left optic neuritis two months post status epilepticus. Spontaneous improvement was seen with GFD and methylprednisolone and complete remission was observed after adding azathioprine.
Hijaz et al. [21]	2014	Case report	A two-year-old male child presented with lethargy and status epilepticus and was diagnosed with celiac crisis. A significant improvement was noted with methylprednisolone and the reintroduction of GFD.
Jorge et al. [22]	2014	Case report	A three-year-old female presented with ataxic gait, dysarthria, and dysphagia was diagnosed with gluten sensitivity. She showed clinical improvement after the introduction of GFD.
Pacitto et al. [23]	2017	Case report	A 23-month-old male presented with a sudden refusal to walk who, on examination, had a loss of b/l LL DTRs. He was diagnosed with celiac disease. After the introduction of strict GFD, his symptoms have completely resolved.
Krom et al. [24]	2017	Case report	A 17-month-old female presented with a rapid progressive unwillingness to walk, sit, and play and complained of vomiting and lack of appetite and was diagnosed to have biopsy-proven CD. Complete recovery of symptoms was noted after management with strict GFD and methylprednisolone. Delayed developmental milestones, methylprednisolone + GFD, improved developmental milestones at one-month follow-up.

Seven studies were found to be case reports, and their findings are discussed separately in the section "Gluten-Free Diet (GFD)". Among the 12 remaining studies, five were cross-sectional [5-10], one was case-control [11], two studies were retrospective cohort [12,13], and the remaining four were prospective studies [14-17]. Of these, eight studies described neurological manifestations and biopsy findings [6-12,17], out of which serological investigations were reported in six studies [6,7,10,11,12,17], EEG findings in four studies [6,7,10,12], and neurological imaging was done in four studies [6,7,10,17]. 

*Results of Individual Studies* 

Table 3 discusses the relevant neuropsychiatric manifestations, serological and biopsy findings, EEG findings, and imaging findings from the selected studies. Sel et al. describe four patients (3.41%) with seizures, one with frontal short-duration headaches and attention-deficit hyperactivity disorder (ADHD), one patient having down phenotype, global developmental delay, and axial hypotonia, a case of optic neuritis and one multifocal leukoencephalopathy that presented with hypersomnia among 117 atypical CD patients with positive serology and biopsy [12]. 

**Table 3:** Clinical manifestations and investigations of individual studies Anti-tTG, Anti-Tissue Transglutaminase; AGA, Anti-Gliadin Antibodies; BG, Basal Ganglia; CD, Celiac Disease; EMA, Endomysial Antibodies; EEG, Electroencephalography; MRI, Magnetic Resonance Imaging; PCC, Posterior Cerebral Calcifications; RLS, Restless Leg Syndrome; TGA, Tissue Transglutaminase antibodies. *Further details of neurological findings have been described in the text.

S.No.	Author	Type of Study	Neuropsychiatric manifestations	Neuroimaging and EEG	Serology/ Genetics (n)	Biopsy/ Autopsy
1	Aksoy et al. (2016) [6]	Cross-sectional study	16/65 CD patients had neurologic abnormalities	MRI - white matter and BG Lesions (1); cerebellar atrophy (1) EEG - generalized (1); focal (did not determine an increased prevalence of RLS but found early onset as an unexpected outcome of the study). 4) epileptiform activity*	EMA+: IgA/IgG (36/5); TGA+: IgA/IgG (32/6); AGA+: IgA/IgG (22/27)	MARSH 1,2 - 15/65; MARSH 3 - 50/65
2	Ferlazzo et al. (2019) [7]	Cross-sectional study	9/60 CD patients with posterior cerebral calcifications and epilepsy	MRI - posterior cerebral calcifications in occipital (5), parieto-occipital (2), and parietal (1) lobes	Anti-TG-6+ (1)	5/9 cases histologically confirmed (ESPGHAN)
3	Işikay et al. (2015) [17]	Prospective study	6/175 CD patients had seizures	EEG - Epileptiform activity originates in TPO (2), TO (1), O (1), TP (1), bilateral synchrony (1) among CD patients	Total serum IgA normal in all cases	MARSH 3 in all six cases
4	Sel et al. (2017) [12]	Retrospective study	8/117 children with neurological abnormalities diagnosed with CD*	MRI - Multifocal leukoencephalopathy (1), asymmetrically dilated TO lobes and lateral ventricle (1)	Anti-tTG IgA above normal levels in all cases	MARSH 3b in all eight cases
5	Swartwood et al. (2021) [10]	Cross-sectional study	25 focal and 31 generalized seizures among 56 CD patients with seizures	EEG - Generalized (11) and focal (15) epileptiform activity among the cases	Anti-tTG + (20)	36 cases biopsy positive

Aksoy et al. (2016), having discovered 16 CD patients with abnormal neurological findings, performed additional neurological investigations such as visually evoked potential (VEP), brainstem auditory evoked response (BAER), and electroneuromyography (ENMG) with parental consent, the results of which are as follows. VEPs showed one unilateral and mild, four bilateral mild, two bilateral marked, six absent, and three normal responses. BAER suggested one sensory-neural, five absent, and 10 normal findings. ENMG revealed four distal axonal neuropathies, two of which were mild, one proximal and distal mild axonal neuropathy, two demyelinating, and one sensory motor axonal neuropathy. Six cases showed absent ENMG and two were normal. While serological tests showed no correlations with neurological findings, 12 out of 16 patients with abnormal neurological signs had stage 3 severe histopathological findings [6]. Isikay et al. (2015) discovered that a more severe histopathological grade (type 3b) is associated with patients presenting with neurological manifestations when compared with those without (type 3a) [8]. In addition, Gungor et al. did not find a statistically significant association between ADHD and CD based on serology [11].

Aksoy et al. (2016) performed tissue typing on 30 patients. HLA DQ2 was positive in six of 11 patients with abnormalities [6]. In another study, by De Leo et al. (2018), all CD patients tested positive for HLA DQ2 or DQ8 and anti-TG2 autoantibodies with a significant difference compared to controls (p = 0.04) [13]. Işikay et al. (2015) further mentioned a significant positive association (Pearson correlation analysis) between tTG levels and epileptiform activity in sleep (p = 0.014) and awake (p = 0.019) EEG [17]. In another study on children with restless leg syndrome (RLS) and CD, Işikay et al. (2018) concluded that although no increased prevalence of RLS was found, early onset of the disease was discovered in CD patients as an unexpected outcome [9].

Gluten-Free Diet (GFD)

Statistically significant findings are seen in five [13-17] of the seven studies that reported the impact of GFD. A p-value ≤0.001 was noted in all of these studies. Overall improvement in the clinical picture [14,16], serological findings [13], and EEG findings [10,12,17] were explicitly described, as seen in Table 4. 

**Table 4:** Impact of gluten-free diet CSHQ, Child's Sleep Habits Questionnaire; EQ5D, EuroQol-5 Dimension; EEG, Electroencephalography; GTS-QOL, Gilles de la Tourette Syndrome-Quality of Life Scale; TG, Tissue Transglutaminase; PSQ, Perceived Stress Questionnaire; YBOCS, Yale-Brown Obsessive-Compulsive Score; YGTTS, Yale Global Tic Severity Score.

S.No.	Study​	Pre-GFD ​	GFD duration​	Post-GFD​	p-Value​
1	De Leo et al. (2018)​ [13]	Anti-TG2 positive: 2; anti-TG6 positive: 4​	24 months ​	Anti-TG2 positive: 2; anti-TG6 positive: 4​	p < 0.001​
2	Işikay et al. (2015)​ [17]	EEG positive: 6​	Variable ​	EEG negative: 6​	-​
3	Rodrigo et al. (2018)​ [14]	YGTTS score = 55±17.5;​ YBOCS score = 15.3 ± 12.3; EQ5D score = 0.62 ± 0.23;​ GTS-QOL score = 42.8 ± 18.5​	1 year​	YGTTS score = 27.3 ± 22.3;​ YBOCS score = 5.4 ± 8.6;​ EQ5D score = 0.88 ± 0.17;​ GTS-QOL score = 22.4 ± 19.9​	p < 0.001​
4	Suzer Gamli and Keceli Basaran (2022)​ [15]	CSHQ total score: 46​	6 months​	CHSQ total score: 40 ​	p < 0.001​
5	Yerushalmy-Feler et al. (2018) [16]​	Case PSQ = 0.17 ± 0.14;​ Control PSQ = 0.26 ± 0.15​	6 months​	Case PSQ = 0.07 ± 0.06​; Control PSQ =0.2 ± 0.11 ​	p < 0.001​
6	Sel et al. ( 2017) [12]​	EEG positive: 5​	Variable​	EEG positive: 1​	p < 0.001
7	Swartwood et al. (2021)​ [10]	Biopsy confirmed CD EEG positive: 17;​ Serologically suggestive CD EEG positive: 7​	Not mentioned​	Biopsy confirmed CD EEG positive: 4;​ Serologically suggestive CD EEG positive: 3​	-​

*Case Reports* 

Seven cases of patients with various presentations of CD and gluten sensitivity were identified [18-24]. Table 5 describes the detailed findings of the case reports. 

**Table 5:** A glance into the case literature AGA, Anti-Gliadin Antibodies; Anti-tTG2, Anti-Tissue Transglutaminase 2; B/L, Bilateral; CRP, C-Reactive Protein; DTR, Deep Tendon Reflexes; EMA, Anti-Endomysial Antibodies; GFD, Gluten-Free Diet; LL, Lower Limbs; MOG, Myelin Oligodendrocyte Glycoprotein; tTG IgA, Tissue Transglutaminase Immunoglobulin A; tTG IgG, Tissue Transglutaminase Immunoglobulin G.

S.No	Authors	Age & Gender	Presentation	Findings	Management	Prognosis
1	Bashir et al. (2019) [18]	16-month-old female	Vomiting and regression of motor skills	⬇ muscle tone and movements. ⬆ tTG IgA and tTG IgG levels. Villous blunting and increased intraepithelial lymphocytes.	GFD	Antibodies normalized in nine months. The patient started walking independently.
2	Boskovic and Stankovic (2014) [19]	15-year-old female	Acute weakness and pricking sensation in lower limbs	⬇ knee and ankle jerk reflexes. ⬇ Pain, touch, and temperature in lower limbs. ⬆ tTG IgA and tTG IgG levels. Sensory-motor demyelinating peripheral neuropathy	Strict GFD	Completely asymptomatic at one-year follow-up.
3	Ching et al. (2015) [20]	Four-year five-month-old female	Left optic neuritis two months post status epilepticus	White matter demyelination. ⬆ CRP. Positive anti-MOG antibodies	Methylprednisolone + azathioprine + GFD	Asymptomatic in the follow-up.
4	Hijas et al. (2014) [21]	Two-year-old male	Lethargy and status epilepticus	Lethargic, postictal status. Generalized hypertonicity. ⬇ DTR. No epileptiform discharges. ⬆ Anti-tTG antibodies. Villous atrophy, intraepithelial lymphocytes, and increased length of crypts (Marsh IIIb to IIIc).	Methylprednisolone + GFD	Complete reversal of neurological function with improved communication and ambulation.
5	Jorge et al. (2014) [22]	Three-year-old female	Ataxic gait, dysarthria, and dysphagia	⬆ tTG IgA levels. Villous atrophy, crypt hyperplasia, and intra-epithelial lymphocytes (MARSH III).	GFD	Normal anti-tTG2 antibody levels and psychomotor development.
6	Pacitto et al. (2017) [23]	23-month-old female	Pale, irritable, and muscle hypotrophy.	Stunting. ⬇ muscle tone, absent B/L LL deep tendon reflexes. ⬆ AGA, tTG levels, EMA levels.	GFD	Overall significant improvement in clinical status.
7	Krom et al. (2017) [24]	17-month-old female	Delayed developmental milestones	Irritable, wasted legs and buttocks. ⬆ tTG IgA and AGA levels. Subtotal villous atrophy and lymphocytic gastritis (MARSH IIIb).	Methylprednisolone + GFD	Improved developmental milestones at one-month follow-up.

In all of the above-mentioned case reports, GFD had a significant impact on improvement in clinical and laboratory findings. In fact, in the case report by Boskovic et al., accidental ingestion of a diet rich in gluten after a period of management with GFD caused the acute episode, which was resolved after resuming strict adherence to GFD [19]. 

Discussion

This review article aimed to explore the association and pathogenesis of gluten sensitivity causing extra-intestinal, primarily neurological manifestations. In children with a higher chance of atypical presentations, it is crucial for physicians to have a low threshold for suspicion since misdiagnosis can result in the prescription of unnecessary and potentially toxic medications. Hence, understanding the pathophysiology, presentations, histologic grading, and effect of GFD is of prime importance and is discussed in detail below. 

Pathophysiology

Based on the available literature, we discuss here the key findings regarding the pathogenesis of common neurological manifestations such as epilepsy, peripheral neuropathy, headache, delay/regression of developmental milestones in children, and ADHD. Epilepsy of unknown origin is the earliest known neurological association of CD in children, commonly presenting as partial (simple/complex) seizures originating in the occipital lobe. Occipital calcifications are commonly associated with HLA DQ2/8, causing epilepsy. Gobbi syndrome or CEC syndrome is a typical association of CD involving posterior cerebral calcifications and occipital lobe seizures [25], which corresponds to our results in Table 3. Our study concludes that seizure activity was seen more frequently in patients with CD as compared to their respective control groups. This is in line with a study by Peltola et al., where a link between epilepsy and CD with a prevalence of 4 to 8% has been reported [25]. 

**Figure 3:**
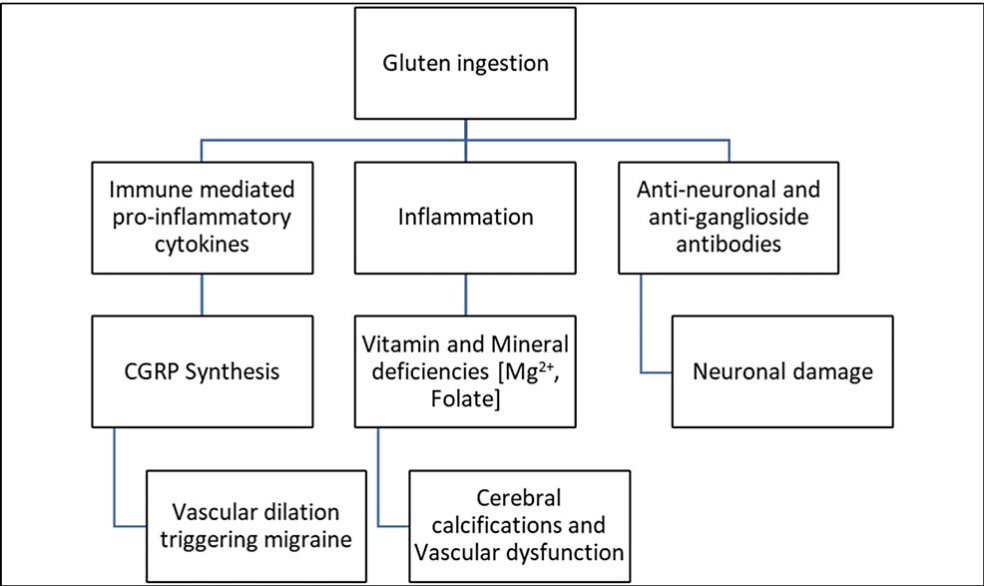
Pathogenesis of headache in celiac disease CGRP, Calcitonin Gene-Related Peptide.

Peripheral neuropathy and demyelinating lesions are common associations reported by Coronel-Rodríguez et al. as being associated with decreased absorption of some dietary components such as tocopherol, cobalamin, or thiamine [2]. In one of our case reports, Boskovic et al. describe the case of a 15-year-old female who presented with acute weakness and pricking sensation in her lower limbs on stopping GFD, which she was on since she was diagnosed with CD at nine months of age [19]. Headache, the most common neurological association in CD children, is hypothesized to be due to several pathogenetic mechanisms, as mentioned in Figure 3 [2]. 

A very early life manifestation of gluten-related pathology in children could be developmental delay or regression of milestones. As seen in Table 5, Krom et al., Bashir et al., and Jorge et al., all mention children with regression of motor skills and developmental delays, which had drastically improved once the children were started on GFD [18,22,24]. Some of their symptoms even resolved completely. This is hypothesized to be due to malabsorption causing deficiency of the micronutrients essential for the development of the central nervous system in children [26].

ADHD is a common psychiatric manifestation, with particular symptoms having a higher association with gluten sensitivity. Sluggish cognitive tempo is a pattern of confusion, inconsistent orientation and alertness, physical underactivity, lack of mental alertness, and daydreaming [27]. While Gungor et al. describe an increase in these presentations among children with CD, their case-control study did not find a statistically significant association between ADHD and CD based on serology [11]. 

ESPGHAN Criteria and MARSH Grading

CD is an immune-mediated enteropathy with primarily intestinal manifestations. Table 3 discusses the neurological manifestations we found, in our literature search, in children with serology and/or biopsy-proven CD, along with imaging and EEG findings. A point of interest in these children is that the symptoms adopt a "non-classic" form, wherein the presenting symptoms are purely neurological without gut symptoms, making its diagnosis difficult [28-30]. This calls for a high index of suspicion. These children should be screened with serological tests and duodenal biopsy for evidence of CD.

The New European Society of Pediatric Gastroenterology, Hepatology, and Nutrition (ESPGHAN) criteria do not require deamidated gliadin peptide antibodies (DGP-IgG/IgA) for initial testing. The presence of significant serum levels of tissue transglutaminase antibodies, particularly anti-TG-2 IgA, and anti-TG-6 IgA antibodies, along with the quantification of serum IgA, constitute serological diagnosis. If total IgA is low/absent, IgG levels are determined.

In patients with parental consent, if the value of TGA-IgA is 10 times more than the upper limit of normal, then a biopsy is not required for diagnosis. In this case, confirmation with endomysial antibodies (EMA-IgA) is required. Even HLA DQ2-/DQ8 and gastrointestinal symptoms are not obligatory criteria for diagnosis. However, in patients with positive TGA-IgA levels 10 times less than the upper limit of normal, a biopsy is required for diagnosis. Four biopsies are required from the distal duodenum, and at least one from the bulb should be taken to provide adequate evidence of CD. Reevaluation in patients with discordant results is required. Those with mild/absent histological changes (MARSH 0/I) but confirmed antibody presence (TGA-IgA/EMA-IgA+) should be monitored closely. For those with concurrent diabetes mellitus (DM) I or IgA deficiency, an intestinal biopsy is mandatory for diagnosis [31].

The histopathological grading for the diagnosis of CD has been highly controversial, and several grading criteria have been proposed so far. Among those, the most important remains to be MARSH grading which is a part of the ESPGHAN criteria. Various modifications of this grading have been outlined in Table 6. In addition, Lonardi et al. suggest an immunostaining method that can be used in latent CD cases where TCR-gamma coupled with CD3 staining can diagnose these unequivocal histologies [32-42]. This is particularly beneficial in the diagnosis of children with neuropsychiatric manifestations.

**Table 6:** Various histopathological classifications of celiac disease **Upper limit of normal in duodenal mucosa. IEL, Intra-Epithelial Lymphocytes.

Marsh 1992 and Rostami et al. 2015 [34-37]	Rostami et al. 1998, 1999 [38,39]	Oberhuber et al. 1999 [40]	Corazza & Villanacci 2005 [41]	Ensari 2010 [42]
Type 0: Microscopic enteritis; normal villi with pathological increase of T cells, altered enterocytes, shortened microvilli with increased α/β/γ/δ T-cell receptors	-	-	-	-
Type 1: Microscopic enteritis: increased IEL count (> 20 IELs per 100 enterocytes)	Marsh I: normal villous epithelium, with increased IEL count (>30 IELs per 100 enterocytes)	Type 1: Infiltrative lesions	Grade A: No signs of atrophy, normal villous architecture (with or without) crypt hyperplasia and IEL count (≥25 IELs per 100 enterocytes)	Type 1: Normal villous architecture with IE lymphocytosis
Type 2: Microscopic enteritis (increased IEL count (>20 IELs/100 enterocytes)) with crypt hyperplasia	Marsh II: Presence of enlarged crypts and influx of inflammatory cells	Type 2: Crypt hyperplasia		
Type 3: Villus effacement with crypt hyperplasia	Marsh IIIa: (partial VA) shortened blunt villi with infiltration of IEL and hyperplastic crypts	Type 3A: Partial villous atrophy	Grade B1: Villous-crypt ratio <3:1 with increased IEL count (>25/100 enterocytes)**	Type 2: Shortened villi (<3:1 or <2:1 in bulbus) with crypt hyperplasia and IE lymphocytosis
	Marsh IIIb: (subtotal VA) atrophic villi (recognizable) with inflammatory cells and hyperplastic crypts	Type 3B: Subtotal villous atrophy		
	Marsh IIIc: (total villous atrophy) total absence of villi, severe atrophic, hyperplastic, infiltrative lesion	Type 3C: Total villous atrophy	Grade B2: No observable villi with completely flat and atrophic mucosa, and IEL count (≥25 IELs per 100 enterocytes)	Type 3: Completely flat mucosa with crypt hyperplasia and IE lymphocytosis
Type 4: Destructive lesion	Not considered	Type 4: Complete destruction of the villi	Not considered	Not considered

*Gluten-Free Diet* 

Nikpour reported an improvement in 90% of the patients upon starting GFD. Their symptoms rapidly regressed, if not resolved completely. On examination, there was normalization of both antibody levels and improvement in small bowel histopathological changes. However, the remaining 10% of patients remained refractory to GFD but responded to other dietary restrictions, including protein, or failed to respond to all types of intervention. The latter group tended to develop further complications of CD, such as T-cell lymphoma. Interestingly, they also note that bowel mucosal changes might not be completely reversed on GFD [43].

Table 4 demonstrates the effect of GFD on neurological symptoms of CD. Studies by De Leo et al. and Rodrigo et al. noted statistically significant improvement in GFD-compliant patients with neurological manifestations of CD [13,14]. Furthermore, Rodrigo et al. noted an overall decrease in disability, motor symptoms, phonics and tics, obsessions, and compulsions [14]. In another study, Sel et al. noted that GFD improved the seizure-free duration by up to 60% in cases. Also, they stated that GFD may be sufficient to maintain this interval without the use of pharmacotherapy [12]. 

## Conclusions

Our systematic review discusses the intricate relationship between neurologic disease and immune-mediated enteropathy. We have clear evidence that several neurological conditions (neuropathy, ADHD, epilepsy, RLS, and developmental delays) have not only a significant association with gluten but can also potentially benefit from GFD. Further research and clinical trials are warranted to implement CD testing and GFD as primary preventive strategies. In conclusion, our review underscores the importance of gluten while dealing with idiopathic neurological conditions in children and hopes to shed light on this commonly misdiagnosed and easily manageable disease.
